# Highly Permeable Ultrafiltration Membranes Based on Polyphenylene Sulfone with Cardo Fragments

**DOI:** 10.3390/polym16050703

**Published:** 2024-03-05

**Authors:** Alisa Raeva, Dmitry Matveev, Nikolay Bezrukov, Evgenia Grushevenko, Azamat Zhansitov, Zhanna Kurdanova, Kamila Shakhmurzova, Tatyana Anokhina, Svetlana Khashirova, Ilya Borisov

**Affiliations:** 1 Center for Progressive Materials and Additive Technologies, Kabardino-Balkarian State University Named after H.M. Berbekov, 360004 Nalchik, Russia; dmatveev@ips.ac.ru (D.M.); azamat-z@mail.ru (A.Z.); kurdanova09@mail.ru (Z.K.); shahmurzova.kamila@yandex.ru (K.S.); new_kompozit@mail.ru (S.K.); 2 Laboratory of Polymeric Membranes, A.V. Topchiev Institute of Petrochemical Synthesis Russian Academy of Sciences, 119991 Moscow, Russia; bezrukov@ips.ac.ru (N.B.); evgrushevenko@ips.ac.ru (E.G.); tsanokhina@ips.ac.ru (T.A.)

**Keywords:** polyphenylene sulfone, polycondensation, ultrafiltration, membranes, coagulation rate, tensile strength, viscosity

## Abstract

For the first time, copolymers of polyphenylene sulfone (PPSU) with cardo fragments of phenolphthalein (PP) were synthesized to develop highly permeable flat-sheet ultrafiltration membranes. By introducing cardo fragments into the polymer chain, we achieved a mechanical strength 1.3 times higher than the strength of commercial PPSU. It is shown that the introduction of the cardo monomer significantly increases the solubility of the polymer in aprotic solvents. The highest solubility is observed at the concentration of PP 50 mol.%. It is found that reduced viscosity of cardo polymer solutions leads to an increase in the coagulation rate. The permeance of asymmetric ultrafiltration membranes increases with PP concentration from 17.5 L/(m^2^·h·bar) (10 mol.% PP) to 85.2 L/(m^2^·h·bar) (90 mol.% PP). These data are in agreement with the results of a study of the coagulation rate of polymer solutions. Thus, for ultrafiltration membranes with 1.5–8 times higher permeance in comparison with PPSU due to the introduction of cardo fragments in the polymer chain, possessing high rejection of the model dye Blue Dextran (M_W_ = 70,000 g/mol), more than 99.2%, as well as high strength characteristics, were achieved.

## 1. Introduction

In the last few decades, the demand for fresh water resources has been increasing by about 1% per year. This phenomenon is caused by such reasons as climate change, the socio-economic development of society, and, to a greater extent, the growth of our planet’s population [1]. In fact, human activities are the main cause of the increasing pollution of fresh water sources by biological wastes, bacteria, and viruses [2]. Traditional water treatment methods, widely used in industry, have limitations. For example, chlorination is a process accompanied by the formation of toxic by-products during the reactions of chlorine with organic compounds that are part of surface water [3]. UV radiation and ozonation are very effective in pathogen control; however, their application is limited by the high cost of such technologies [4,5].

Today, the attention of specialists is attracted to membrane technologies as they combine the low cost of the process, compactness, and environmental friendliness due to the lack of formation of harmful by-products in the process of water treatment [4,6]. Of the wide range of membrane separation technologies, micro- and ultrafiltration are the most effective in purifying water from bacteria and viruses [5,7]. However, there is one very important requirement for the membrane element, which is sterilization capability.

Currently, polymer membranes based on polysulfone (PSF), polyethersulfone (PES), polyvinylidene fluoride, and polyamide are leading in the market as they are simple and cheap, as well as easily scalable [7,8,9]. Among the many proposed polymers, polysulfones are relatively resistant to the high-temperature sterilization of superheated steam. However, PSF can withstand the sterilization process no more than 100 times without losing its mechanical properties [10].

From the point of view of increased stability, one promising polymer in the range of polysulfones is polyphenylene sulfone (PPSU). It has the highest indices of thermal, chemical, and hydrolytic stability, as well as mechanical strength. However, a number of factors limit the forming of membranes by the phase-inversion method: limited solubility of the polymer in aprotic solvents due to the presence of Cl-terminal groups, as well as its hydrophobicity. It was shown in a previously published result that it is difficult to form membranes based on pure commercial PPSU due to the low molecular weight of the commercial grades of these polymers produced [11].

A literature review has shown that research in the field of the application of PPSU as a membrane material comes down to the adaptation of commercial grades with close molecular weight through its modification and design of hybrid materials based on it [11]. Summarizing the information, the main directions include sulfonation, blending with a more hydrophilic membrane-forming polymer, introduction of nanoparticles into the polymer solution, and introduction of hydrophilic and oligomeric additives into the polymer solution.

The sulfonation of PPSU leads to a significant increase in membrane hydrophilicity, membrane permeance, and antifouling properties [12,13,14] but decreases mechanical strength [15]. The effect of the degree of sulfonation on the morphology and performance of membranes has been studied [12,15]. It was found that sulfonated PPSU polymers (s-PPSU) have a larger homogeneity region in the ternary phase diagram “s-PPSU-solvent-water” and require more water to initiate phase decomposition [15]. This leads to slower phase separation and a lower phase inversion rate compared to PPSU [15]. It has been shown that polymer swelling can reduce water flux through the membrane when the degree of sulfonation is high. Such polymers form membranes with a low content of macrovoids [12,15]. Sulfonated PPSU is used as a support material for composite membranes used in direct osmosis, nanofiltration [16,17,18,19,20], membrane gas separation [21], pervaporation [22], ion exchange membranes [23], and fuel cell membranes [23,24,25,26,27].

The polymer blend is a widely used approach to membrane modification because of its simplicity and commercial attractiveness. Membranes made of blends of PPSU/polyetherimide (PEI) [28] and PPSU/PSF [29] for ultrafiltration, PPSU/polyimide for nanofiltration of organic solvents, and PPSU/(copolymer of poly (bisphenol A-co-4-nitrophthalic anhydride-co-1,3-phenylenediamine)) for gas separation [30] are known. In [28], flat-sheet UF membranes were prepared from solutions containing a mixture of DMF and PPSU in different ratios (40:0 to 0:40). PEG-200 was used as a pore-forming additive. It was shown that the introduction of PEI into the PPSU solution increased membrane hydrophilicity and surface roughness, which, according to the authors, prevented adsorption on the membrane surface and increased fouling resistance [28]. To increase the membrane performance during water treatment, activated carbon was added to the polymer solution containing a mixture of PPSU, PEI, and PEG [31]. The addition of carbon particles and PEG to the polymeric membranes has increased the flux of the composite membranes and improved the resistance of the membrane to clogging during the filtration process. The best component ratio of the activated carbon/PPSU/PEI/PEG composite membrane was 0.25:35:5:6 wt.%. The water flux through the membrane and humic acid rejection were 184 L/m^2^·h and 80%, respectively. Moideen et al. [29] investigated a mixture of PSF and PPSU for the fabrication of hollow fiber membranes for the removal of heavy metals from water. PEG-1000 was used as a pore-forming and hydrophilic additive to the polymer solution. The addition of PEG-1000 to the polymer solution increased the hydrophilicity of the blended membranes (wetting angle decreased to ~65°), which increased the membrane permeance and antifouling properties. It was shown that the membrane with a PPSU:PSF ratio of 75:25 had the best performance. The rejections of heavy metal ions during ultrafiltration in the form of their complexes with polyethylenimine were 99.5% and 95.5% for Pb^2+^ and Cd^2+^, respectively.

A review of the literature indicates that the methods used to modify industrial PPSU can achieve significant increases in the permeance and rejection of filtration membranes. 

However, it should be noted that chemical modification of ready-made PPSU, despite its efficiency, requires the use of additional reagents and purification of the new materials, which increases its cost and negatively affects the environment. The introduction of other polymers into the PPSU matrix reduces the membrane resistance to aggressive influences, such as steam sterilization, and negatively affects the mechanical properties of the membranes. It should be emphasized, that the decrease in mechanical properties of modified PPSU is highly undesirable for its membrane application. The use of heterogeneous materials that tend to delaminate cannot ensure the mechanical strength and stability of the membrane over time.

To give the membrane material the necessary properties, such as mechanical strength, hydrophilicity, and increased solubility in solvents required for membrane forming, optimization of the chemical structure at the stage of its synthesis is effective [11,32]. 

One of the proposed solutions to the problems of polymer hydrophobicity and solubility is the introduction of rigid and more polar (cardo) monomers into the main polymer chain during polycondensation. Such modification contributes to the increase in the heat and thermal stability of polymers and also increases solubility in organic solvents due to the reduction in the interchain interaction [33,34]. 

In the present work, the task was to synthesize PPSUs with different contents of cardo fragments, form ultrafiltration membranes based on them, and study the influence of the ratio of cardo monomer on membrane structure, as well as surface, mechanical, and filtration properties.

## 2. Materials and Methods

### 2.1. Materials

N-methyl-2-pyrrolidone (NMP, Chimmed, Moscow, Russia), dimethylacetamide (DMAc, Chimmed, Moscow, Russia), dimethylformamide (DMF, Chimmed, Moscow, Russia), and dimethyl sulfoxide (DMSO, Chimmed, Moscow, Russia) were used as solvents in the preparation of polymer solutions. Polyethylene glycol (PEG, M_W_ = 400 g/mol, Acros Organics, Waltham, MA, USA) was used as a pore-forming additive and distilled water was used as a coagulant. The separation characteristics of flat-sheet asymmetric membranes were investigated using an aqueous solution of model dye Blue Dextran (Sigma-Aldrich, St. Louis, MO, USA) with a molecular weight of 70,000 g/mol. The PPSU and cardo copolymers were synthesized according to the procedure given in the work [33]. The monomer ratios during the synthesis of PPSU and cardo copolymers are presented in Table 1.

### 2.2. Gel Permeation Chromatography (GPC) Method

A Waters GPC system equipped with a differential refractometer (Chromatopack Microgel-5) was used for polymer analysis [11]. The flow rate was 1 mL/min. Chloroform was used as an eluent. The molecular weight characteristics were calculated according to the standard methodology with respect to standard monodisperse polystyrene particles.

### 2.3. Determination of Coagulation Values

The coagulation values (CVs, g/dL) of distilled water for the initial PPSU and its cardo copolymers in NMP were determined as the quantity (g) of water needed to cause the phase separation of 100 mL (1 dL) of 2 wt.% polymer solution by the titration method (T = 23 °C). Coagulation was observed visually. The cloud point was recorded if, after the next addition of titrant, the solution did not become homogeneous within 24 h. CVs are expressed as the weight (g) of the non-solvent per 1 dL of 2 wt.% polymer solution.

### 2.4. Preparation of Dense Polymer Films

Solutions of initial PPSU and cardo copolymers in NMP with a concentration of polymer 20 wt.% were prepared. Dense polymer films were cast by pouring the solution into a Petri dish, the subsequent evaporation of the solvent in a desiccator at a temperature of 50 °C for 2 weeks, and then being kept above the glass transition temperature in a drying cabinet (250 °C) for 1 day.

### 2.5. Investigation of Mechanical Properties of Dense Polymer Films

To investigate the mechanical properties of dense polymer films, a TT-1100 tensile testing machine (Cheminstruments, Fairfield, CT, USA) was used [35]. The traverse speed during the tests was 3.8 cm/min. The studies were carried out at room temperature (21–23 °C). The length of the samples was 70 mm. Young’s modulus values were determined as the slope of the initial section of the stress–strain diagram.

### 2.6. Preparation of Casting Solutions

The casting solutions of the following compositions were prepared in the course of the work (Table 2):

Two-component solutions were prepared with a polymer/solvent ratio—20/80 wt.%. For three-component solutions, the composition of polymer/solvent/pore-forming agent 20/50/30 wt.% was used.

### 2.7. Determination of Dynamic Viscosity of Casting Solutions

The dynamic viscosity of polymer solutions was measured using an Anton Paar MCR 72 modular rheometer [11]. The measurements were conducted at a fixed temperature of 23 °C.

### 2.8. Kinetics of Coagulation of Casting Solutions

To study the kinetics of the coagulation of PPSU and cardo copolymers solutions, the “limited” layer technique was used [36]. The method used allows us to reproduce the process of porous structure formation in an asymmetric polymer membrane. The coagulation kinetics of PPSU and cardo copolymers were evaluated as the rate of coagulation of a layer of polymer solution of a certain thickness. The rate was calculated as the ratio of the thickness of the polymer solution layer to the time of its coagulation. The rate of the coagulation front was averaged over five measurements. Distilled water was used as a coagulant.

### 2.9. Preparation of Flat-Sheet Asymmetric Membranes

The prepared casting solutions of the initial PPSU and cardo copolymers were applied to the glass plate with a thickness of 200 µm. After application on the glass plate, the solution was immersed in a coagulation bath (distilled water) for 24 h. After coagulation, the membrane was washed with distilled water and kept in a new portion of distilled water for 24 h to wash out the solvent residues.

### 2.10. Investigation of Transport and Separation Properties of Flat-Sheet Asymmetric Membranes

Transport and separation characteristics of the membranes were investigated in the dead-end stirred filtration cell. In order to reduce the effect of concentration polarization during filtration, the solution was continuously stirred over the membrane using a magnetic stirring device (MR Hei-Mix S, Heidolph, Schwabach, Germany). The area of the membrane was 7.9 cm^2^. The volume of liquid poured into the cell was 900 mL. For the initial permeance evaluation, distilled water was used as the filtered liquid. To evaluate the separation properties, an aqueous solution of the model dye Blue Dextran (Sigma-Aldrich, St. Louis, MO, USA, M_W_ = 70,000 g/mol) with a substance concentration of 100 mg/kg was used. The value of transmembrane pressure during filtration was equal to 7 bar. Filtration was carried out for all samples until the volume of the permeate reached 90% of the initial volume of the water and 50% of the initial volume of the model dye solution.

The permeance (P, L/(m^2^·h·bar)) of flat-sheet membranes was calculated by Equation (1):

(1) $$P=\frac{V}{S\cdot t\cdot ∆p}$$

where *V* is the volume of the selected sample (L), *t* is the sampling time (h), *S* is the surface area of the selective layer of the flat-sheet membrane (m^2^), and Δ*p* is the overpressure (bar).

The retention (*R*, %) of the model dye Blue Dextran was calculated using Equation (2):

(2) $$R=\frac{1-C_{p}}{C_{f}}\cdot 100\%$$

where *C_p_* is the concentration of the solute in the permeate (mg/L) and *C_f_* is the concentration of the solute in the feed stream (mg/L).

### 2.11. Scanning Electron Microscopy (SEM) Method

In order to study the porous structure of the obtained membranes, the SEM method was used. SEM photographs were obtained using a Thermo Fisher Phenom XL G2 Desktop SEM unit (Waltham, MA, USA). Cross-sections of the membranes were obtained by successive operations: impregnating the samples in 2-propanol and breaking them in a liquid nitrogen environment. Then, a thin layer of silver (5–10 nm) was deposited on the samples using a Cressington 108 auto Sputter Coater (Rassendale, Liverpool, UK) in a vacuum cabinet. The accelerating voltage during imaging was 15 keV [35].

## 3. Results

### 3.1. Properties of Polyphenylene Sulfone Cardo Copolymers

#### 3.1.1. Molecular Weight Characteristics of Synthesized Polymers

**Figure 1:**
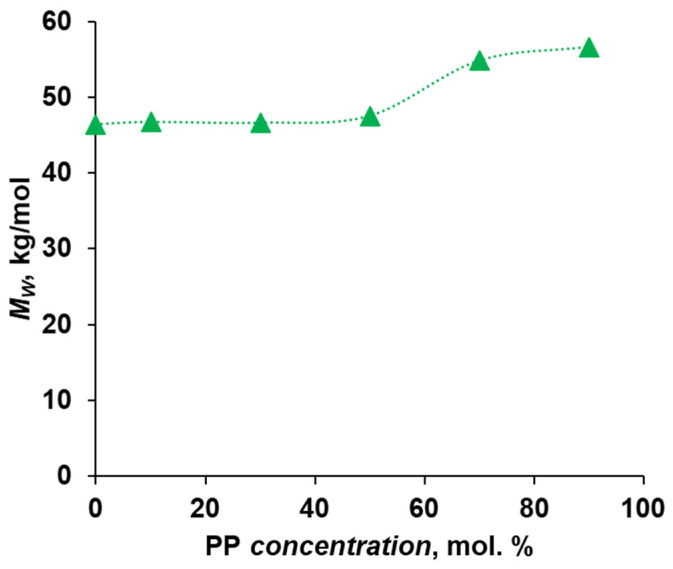
Dependence of the copolymer molecular weight on the concentration of the cardo fragment.

During the study of the molecular weight characteristics of the synthesized polymers and copolymers, it was shown (Figure 1) that with increasing concentration of phenolphthalein (PP) in the reaction mixture, the weight average molecular weight of the polymer increases from 46.4 kg/mol at 0 mol.% phenolphthalein to 56.6 kg/mol at 90 mol.% phenolphthalein. This is probably due to the increase in the stiffness of the polymer chain with an increasing proportion of the cardo fragment. This may lead to a more elongated conformation of the polymer chain and an increase in the size of polymer coils, which is expressed in the apparent increase in the molecular weight of the polymer measured by the GPC method.

#### 3.1.2. Solubility of Cardo Copolymers in Aprotic Solvents

To evaluate the possibility of preparing casting solutions based on synthesized polymers, we have prepared mixtures of the PPSU and PPSU-PP with cardo fragments in NMP, DMAc, DMF, and DMSO with a polymer concentration of 20 wt.% close to the concentration of the casting solution. According to the results in Table 3, it was found that the introduction of PP cardo fragments improves the solubility of PPSU in all the presented aprotic solvents relative to the initial PPSU. Moreover, the best result is achieved at a PP concentration of 50 mol.%. Such a polymer dissolves perfectly in all investigated aprotic solvents. When the concentration of cardo monomer increases up to 90 mol.%, the solubility decreases in solvents such as DMAc, DMF, and DMSO. Nevertheless, both in the case of PPSU homopolymers and for copolymers, NMP is the best solvent. Therefore, the NMP was chosen to form membranes based on the synthesized polymers and to compare their physicochemical and transport properties.

**Figure 2:**
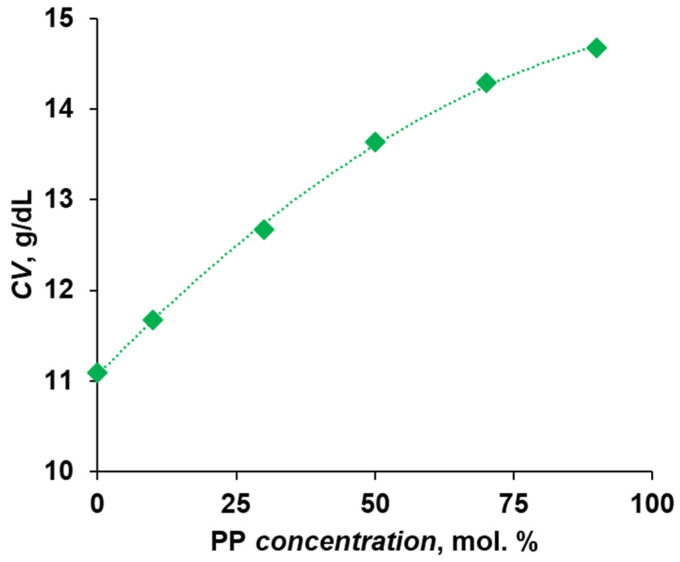
Dependence of the CV on cardo monomer concentration.

In order to compare the stability of polymer solutions in NMP, the dependence of coagulation values (CVs) on the PP concentration in the polymer was found (Figure 2). It was shown that when increasing the concentration of cardo fragments from 0 to 90 mol.%, the CV increases from 11.1 g/dL to 14.7 g/dL, i.e., more water is required for the initialization of phase decomposition. This circumstance also confirms the fact that the solubility of copolymers increases due to the introduction of bulk cardo phenolphthalein fragments. It was previously shown that the introduction of rigid bulk fragments into the main chain of the polymer significantly increases the solubility of polyarylenes [34,37], which was confirmed in the present study.

#### 3.1.3. Mechanical Properties of Flat-Sheet Dense Films

**Figure 3:**
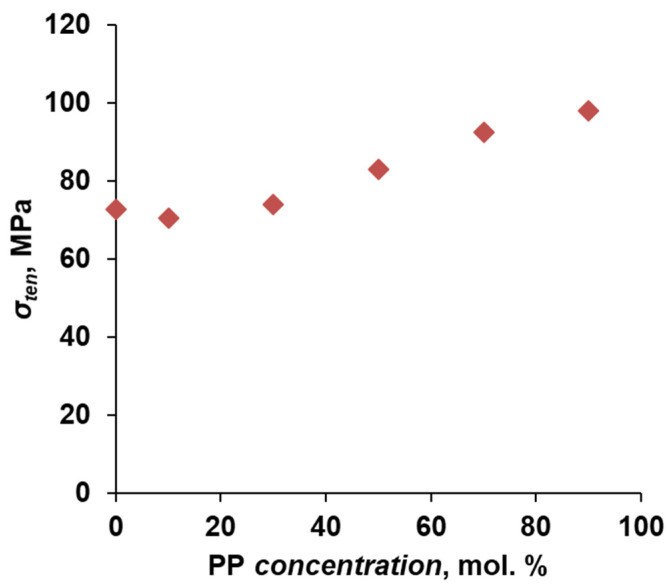
Dependence of the tensile strength of flat-sheet dense films on the concentration of cardo monomer.

Tensile strength is one of the key characteristics when selecting a membrane polymer since the transmembrane pressure in filtration processes can exceed 50 bar [38]. Therefore, the mechanical properties of polyphenylene sulfone copolymers were investigated in this study. The results are presented in Figure 3. No significant changes in tensile strength were observed at a PP concentration of 0–30 mol.%. At the same time, the introduction of more than 30 mol.% of cardo monomer leads to an increase in the strength characteristics of the presented polymeric materials. An increase in strength from 73 to 97.9 MPa was achieved when increasing the concentration of PP from 30 to 90 mol.%.

The data on the strength characteristics of the polymers of a range of commercial polysulfones are given in Table 4. It is shown that in the series of polysulfones, PPSU has high mechanical characteristics, not yielding to the tensile strength of PSU and PES [39,40,41]. It should be noted that the initial PPSU synthesized within this work corresponds to the commercial sample in its properties. At the same time, due to the introduction of cardo fragments into the polymer chain, the mechanical strength is 1.3 times higher than that of the commercial-grade PPSU. Thus, the proposed modification of the side chain will increase the resistance of membranes to the filtered flow pressure.

### 3.2. Investigation of Properties of Casting Solutions Based on PPSU and Cardo Copolymers

**Figure 4:**
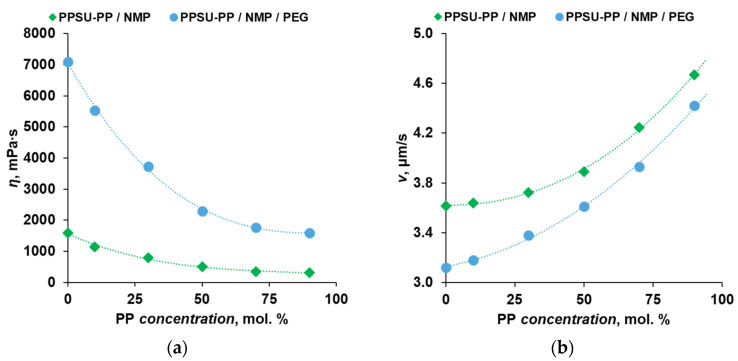
Properties of solutions of initial PPSU and cardo copolymers in NMP: (**a**) dependence of dynamic viscosity and (**b**) coagulation rate of polymer solutions on the concentration of the cardo monomer.

In the work, casting solutions based on the initial PPSU and its cardo copolymers were investigated. As it was shown earlier, the viscosity of the casting solution cardinally influences the coagulation kinetics and membrane morphology [11,36]. Therefore, the dependence of the dynamic viscosity of polymer solutions on the concentration of the cardo monomer in the copolymer based on experimental data was plotted (Figure 4a). It was shown that with increasing concentration of the PP monomer, the dynamic viscosity of two-component 20 wt.% solutions in NMP decreases from 1600 mPa·s at 0 mol.% PP to 305 mPa·s at 90 mol.% PP. The decrease in the viscosity of such solutions with identical composition is a consequence of the increase in the solubility of copolymers in NMP with an increase in the concentration of cardo fragments (Figure 2).

In order to increase the porosity and permeance of the membranes, a pore-forming additive polyethylene glycol (PEG), having a molecular weight M_W_ = 400 g/mol, was introduced into the casting solution in an amount of 30 wt.% [42]. The effect of the additive on the viscosity of the solutions is also presented in Figure 4a. It is shown that the viscosity of solutions containing PEG decreased from 7100 to 1590 mPa·s when the concentration of PP was increased from 0 to 90 mol.%. Thus, PEG increased the solution viscosity by 4.4–6.0 times.

A consequence of the rheological characteristics of the casting solutions is the kinetics of coagulation in the systems PPSU-PP/NMP and PPSU-PP/NMP/PEG. The experimental results are shown in Figure 4b. It can be seen that as the concentration of the cardo monomer increases from 0 to 90 mol.%, the coagulation rate of the solutions increases, in the case of PPSU-PP/NMP solutions, from 3.61 to 4.67 μm/s, and, for PPSU-PP/NMP/PEG solutions, from 3.12 to 4.42 μm/s. The general trend of the increasing coagulation rate is explainable in terms of solution viscosity. The lower the solution viscosity, the higher the diffusion coefficient of the coagulant in the polymer solution, and the higher the coagulation rate [43].

### 3.3. Investigation of Morphology, Transport, and Separation Properties of Flat-Sheet Asymmetric Membranes Based on PPSU and Cardo Copolymers

Flat-sheet asymmetric membranes with the addition of the pore-forming component PEG were cast based on prepared solutions of PPSU and cardo copolymers. SEM images of the membrane cross sections, as well as photos taken when studying the kinetics of coagulation under an optical microscope, are given in Table 5. It is shown that the structures obtained by the optical microscopy are quite similar to the SEM pictures of the membranes. Due to the introduction of a pore-forming agent, we achieved a developed finger-like structure for all membrane samples. It should be noted that with the increasing concentration of cardo fragments from 0 to 90 mol.%, an increase in the ratio of finger-like pores in the support layer of flat-sheet membranes was observed.

**Figure 5:**
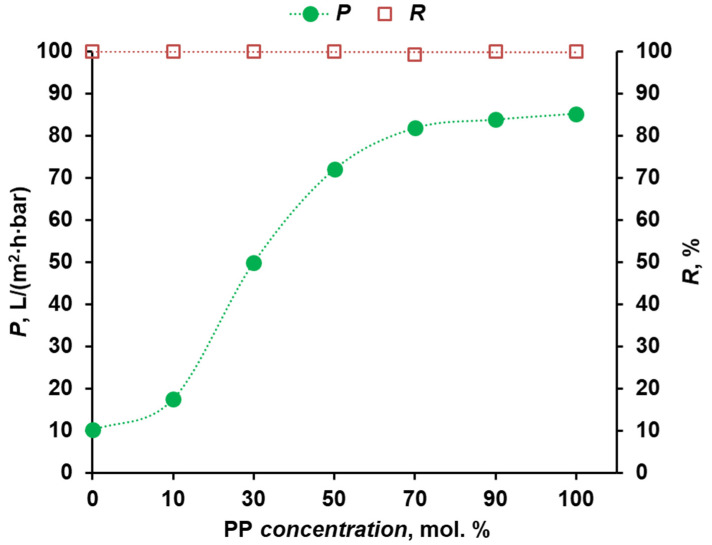
Dependence of transport and separation properties of flat-sheet asymmetric membranes on the concentration of cardo monomer.

The results of studying the filtration properties of membranes were of the greatest importance in the work. Water permeance and Blue Dextran rejection during dye solution filtration are presented in Figure 5. It is shown that the permeance of the sample based on the homopolymer PPSU was equal to 10.4 L/(m^2^·h·bar). The dependence of permeance on the concentration of the cardo monomer increased as the proportion of PP increased from 17.5 L/(m^2^·h·bar) (10 mol.% PP) to 85.2 L/(m^2^·h·bar) (90 mol.% PP). These data are in agreement with the results of a study of the coagulation rate of polymer solutions. It was found that there is a clear correlation between the coagulation rate and the permeance of the liquid through the membrane. Diffusion of coagulant and solvent molecules during phase inversion, as well as mass transport during membrane filtration, will be mainly confined to the subsurface layer, which has the narrowest porous structure. The more a permeable thin porous skin layer is formed on the membrane surface, the faster the coagulation process occurs [11,36]. Consequently, despite the greater stability of the casting solution, the relatively low viscosity of cardo polymer solutions leads to an increase in the permeance of the selective layer of the membrane. It is worth noting that all the studied membranes had a stably high rejection of model dye Blue Dextran (M_W_ = 70,000 g/mol)—99.2–99.9%.

Thus, in this work, for the first time, a highly permeable ultrafiltration membrane based on the PPSU carded copolymer was developed, which has 1.5–8 times greater water permeance, as well as increased mechanical strength compared to a homopolymer membrane and a rejection of the model substance Blue Dextran 99.9%.

## 4. Conclusions

For the first time, copolymers of polyphenylene sulfone (PPSU) with cardo fragments of phenolphthalein (PP) were synthesized to develop highly permeable flat-sheet ultrafiltration membranes. Tests of the mechanical properties of the material demonstrated an increase in strength from 73 to 97.9 MPa with an increase in the PP concentration from 30 to 90 mol.%. At the same time, by introducing card fragments into the polymer chain, we achieved a mechanical strength 1.3 times higher than the strength of commercial PPSU.

The study of the rheological properties of casting solutions showed that the introduction of the cardo monomer significantly increases the solubility of the polymer in aprotic solvents. Moreover, the highest solubility is observed at the concentration of PP 50 mol.%. It is shown that with the increasing concentration of PP monomer, the dynamic viscosity of two- and three-component polymer solutions in NMP decreases more than five times when the PP concentration in the polymer is 90 mol.%. At the same time, the introduction of the hydrophilizing additive PEG increases the solution viscosity by 4.4–6.0 times, which is a positive effect from the point of view of membrane forming.

It is found that as the concentration of the cardo monomer increases from 0 to 90 mol.%, the solution coagulation rate increases in the case of PPSU-PP/NMP solutions from 3.61 to 4.67 μm/s and, for PPSU-PP/NMP/PEG solutions, from 3.12 to 4.42 μm/s. Reduced viscosity of cardo polymer solutions leads to an increase in the non-solvent diffusion coefficients and, as a consequence, an increase in the coagulation rate.

The permeance of asymmetric ultrafiltration membranes increased with PP concentration from 17.5 L/(m^2^·h·bar) (10 mol.% PP) to 85.2 L/(m^2^·h·bar) (90 mol.% PP). These data are in agreement with the results of a study of the coagulation rate of polymer solutions. It was found that there is a clear correlation between the coagulation rate and the permeance of the liquid through the membrane.

Thus, in this work, for the first-time ultrafiltration membranes with 1.5–8 times higher permeance in comparison with PPSU due to the introduction of cardo fragments, possessing high rejection of the model dye Blue Dextran (M_W_ = 70,000 g/mol), more than 99.2%, as well as high strength characteristics, were achieved.

## Figures and Tables

**Table 1:** The monomer ratios during the synthesis of PPSU and cardo copolymers.

PP Concentration, mol.%	PP:DHBP:DCDPS *
0	0:48.30:51.70
10	4.83:43.47:51.70
30	14.49:33.81:51.70
50	24.15:24.15:51.70
70	33.81:14.49:51.70
90	43.47:4.83:51.70

* PP—phenolphthalein, DHBP—4,4′-dihydroxy biphenyl, DCDPS—4,4′-dichlorodiphenyl sulfone.

**Table 2:** Compositions of casting solutions.

PP Concentration, mol.%	Solvent	Additive	C_add_, wt.%
0	NMP	-	-
	DMAc		
	DMF		
	DMSO		
50	NMP		
	DMAc		
	DMF		
	DMSO		
90	NMP		
	DMAc		
	DMF		
	DMSO		
0	NMP	-	-
		PEG	30
10		-	-
		PEG	30
30		-	-
		PEG	30
50		-	-
		PEG	30
70		-	-
		PEG	30
90		-	-
		PEG	30

**Table 3:** Solubility of PPSU and cardo copolymers in aprotic solvents.

Solvent	PP 0 mol.%	PP 50 mol.%	PP 90 mol.%
NMP	+	+	+
DMAc	−	+	+−
DMF	−+	+	+−
DMSO	−	+	−+

«+»—completely soluble, «+−»—opalesce, «−+»—a small amount of sediment is present, «−»—insoluble.

**Table 4:** Mechanical characteristics of commercial grades of polysulfones.

Polymer	Manufacture	Tensile Strength, MPa	Ref.
PPSU Radel R-5100	Solvey, Belgium	73	[39]
PSU Udel-1700	Solvey, Belgium	74	[40]
PES Ultrason E6020	BASF, Germany	30.8	[41]

**Table 5:** SEM photographs of lateral spalling of membranes and structures obtained using an optical microscope in the study of coagulation kinetics.

PP Concentration, mol. %	SEM	Optical Microscope
0	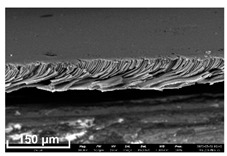	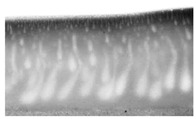
10	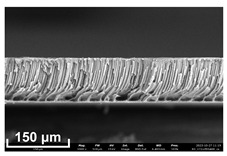	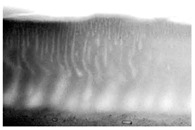
30	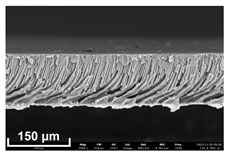	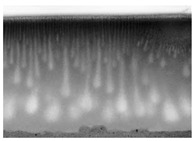
50	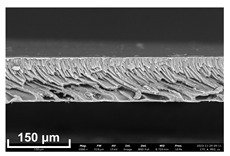	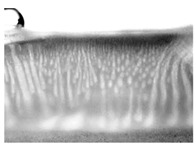
70	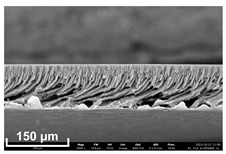	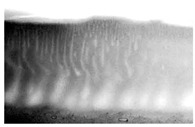
90	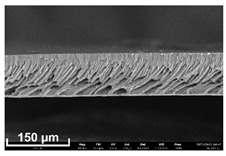	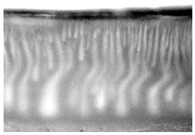

## Data Availability

Data are contained within the article.
